# Epidemiological Characteristics and Evolutionary Characterization of Human Metapneumovirus in Jiaxing, China

**DOI:** 10.3390/v18090978

**Published:** 2026-09-04

**Authors:** Yamei Zhou, Yanqian Wu, Peiyan He, Yong Yan, Ganglin Ren, Xiaofei Zhang, Yin Song, Guoyin Zhu

**Affiliations:** 1 Jiaxing Key Laboratory of Pathogenic Microbiology, Jiaxing Center for Disease Control and Prevention, No. 486, Wenqiao Road, Jiaxing 314050, China; 2 Jiaxing Nanhu District Center for Disease Control and Prevention, No. 370, Zhonghuan South Road, Jiaxing 314050, China

**Keywords:** human metapneumovirus, respiratory virus, genotype, epidemiology, molecular genetics

## Abstract

Human metapneumovirus (hMPV) represents a leading cause of both upper and lower respiratory tract infections among children and adults globally. To investigate the prevalence and evolution of hMPV in the Jiaxing area of China between 2023 and 2025, we screened 3600 pharyngeal swab specimens by real-time PCR, identified 101 positives, and obtained genomic sequences of 48 viral isolates using high-throughput sequencing. Using the sequencing data, we reconstructed a phylogenetic tree and examined amino acid substitutions. The epidemiological analysis revealed an overall hMPV positivity rate of 2.81% (101/3600) in Jiaxing during 2023–2025. Although positive cases were detected across all age groups, they were mainly children, with no significant difference between genders. Regarding seasonal patterns, the peak of hMPV activity occurred predominantly during winter and spring. Over the study period, four genotypes co-circulated, in the order of B2 (41.67%), A2.2.2 (37.50%), A2.2.1 (16.67%), and B1 (4.17%). Further phylogenetic analysis showed that the B1 strains from Jiaxing clustered primarily with those from Beijing, China, while B2, A2.2.1, and A2.2.2 strains were more closely related to strains from the United States and Beijing. Of note, an A2c_111nt-dup_ variant was identified in Jiaxing in 2023. Starting from November 2024, the prevailing genotype transitioned from A2.2.1/A2.2.2 to B2, and B2 emerged as the absolutely dominant strain by 2025. In comparison with earlier circulating strains, several amino acid substitutions have accumulated in current isolates, such as T223N, D280N, I392T, R396Q, S444N, K450R, and T521A in the F protein of B2 strains. Furthermore, the G, L, P, and SH proteins also displayed temporally patterned amino acid replacements. While the biological significance of these mutations is yet to be determined, these results highlight the public health importance of ongoing hMPV surveillance and dynamic monitoring of its genetic evolution.

## 1. Introduction

Human metapneumovirus (hMPV) was first reported in the Netherlands in 2001 and is an important pathogen causing upper and lower respiratory tract infections in children and adults, with its transmission history traceable back to the 1950s [1]. The clinical presentation of hMPV infection is predominantly characterized by symptoms such as cough, dyspnea, and wheezing. Severe infections can progress to bronchiolitis and pneumonia, often presenting with systemic symptoms like fever, and are capable of inducing life-threatening complications, particularly in infants, the elderly, and immunocompromised patients [2]. HMPV is a respiratory pathogen with worldwide distribution, predominantly impacting the pediatric population under 5 years old, and is recognized as a major etiologic agent contributing to the global burden of acute respiratory tract infections (ARTIs) [3,4]. While the highest incidence of infection is observed in infants and young children, infection in the elderly population (especially individuals ≥65 years of age) is associated with a more significant healthcare burden and worse clinical outcomes. According to surveillance data estimates, there were approximately 473,000 hMPV-associated hospitalizations among the elderly globally in 2019, and the actual number of hospitalizations may be higher due to surveillance limitations [5].

The incidence of hMPV exhibits geographic variation, with detection rates among ARTIs cases consistently estimated to range from 5.5% to 25% across different surveillance studies [6,7,8]. Even within the same region, the incidence of hMPV may vary from year to year [9]. According to data from the Chinese Center for Disease Control and Prevention (China CDC), the positivity rate of hMPV in ARTIs from 2009 to 2019 was 4.1%, which is significantly lower than that of influenza virus at 28.5% [10]. However, significant geographical disparities result in non-uniform incidence rates of hMPV among Chinese cities. According to the latest research, the positivity rate of hMPV in some Chinese cities has increased to a range of 4.94% to 9.1% [11,12,13,14].

HMPV is an enveloped, negative-sense single-stranded RNA virus belonging to the family Pneumoviridae and the genus Metapneumovirus [15]. The HMPV genome is approximately 13 kb in length, contains eight genes, and encodes nine proteins: nucleoprotein (N), phosphoprotein (P), matrix protein (M), fusion protein (F), matrix-2 proteins (M2-1 and M2-2), small hydrophobic (SH) protein, glycoprotein (G), and the large (L) polymerase protein [16]. These viral proteins are responsible for diverse functions. Based on the genetic features of the F and G genes, HMPV strains are classified into four genotypes (A1, A2, B1, and B2) and further subdivided into six lineages (A1, A2a, A2b, A2c, B1, and B2) [17,18]. Although all these HMPV genotypes have been reported in China, the proportion of infections caused by each genetic subtype varies across different cities.

After the COVID-19 pandemic, human respiratory infectious diseases have received more attention. Despite continuous research efforts, the comprehensive epidemiological characteristics and genetic diversity of hMPV in China have not been fully elucidated. Jiaxing is an important hub in China’s Yangtze River Delta region with a substantial transient population and significant commercial activity, including one of the largest fruit wholesale markets in East China. After the COVID-19 pandemic, Jiaxing has implemented surveillance for multiple respiratory pathogens, providing unique opportunities for hMPV research. Therefore, this study utilized 2023–2025 surveillance data to evaluate the epidemiological and genetic characteristics of hMPV in Jiaxing, aiming to gain deeper insights into its genetic background and evolutionary status and to promote the epidemiological investigation, prevention, and treatment of hMPV.

## 2. Materials and Methods

### 2.1. Patients and Clinical Samples

From January 2023 to December 2025, throat swabs were collected from influenza-like illness (ILI) cases at Jiaxing First Hospital, a sentinel hospital. A case of ILI was defined as an individual presenting with measured fever (≥38 °C), cough, and symptom onset within the preceding 10 days [19]. Throat swab samples were collected in non-inactivated viral transport media (Yocon MT0901-3, Beijing, China) and transported to the laboratory at 4 °C for testing subsequent molecular analysis. Chi-square tests were used to assess gender differences, and Pearson’s chi-square test was employed to evaluate gender distribution across different age groups.

### 2.2. RNA Extraction and Quantitative Reverse Transcription-Polymerase Chain Reaction

Total RNA was extracted from 200 μL pharyngeal swab samples using an automated nucleic acid extraction system (Tianlong Genrotix 96, Xi’an, China). Detection of common respiratory viruses was performed using a real-time fluorescent quantitative PCR kit (X-abt D404AAYF, Beijing, China), including HMPV, influenza A virus, influenza A H1 subtype, influenza A H3 subtype, influenza B virus, human adenoviruses, human parainfluenza virus, respiratory syncytial virus, enteroviruses and rhinoviruses, human coronaviruses, and human bocavirus. A real-time quantitative PCR kit (X-abt D580MYH, Beijing, China) was used to detect common bacteria, including *Mycoplasma pneumoniae*, Group A *Streptococcus*, *Bordetella pertussis*, *Streptococcus pneumoniae*, *Haemophilus influenzae*, *Legionella* spp., *Klebsiella pneumoniae*, *Aspergillus* spp., *Cryptococcus* spp., *Pneumocystis* spp., *Mycobacterium* spp., *Chlamydia psittaci*, and *Chlamydia pneumoniae*. The kit includes both negative and positive controls, and the PCR procedure was performed according to the manufacturer’s instructions. Samples with a cycle threshold (Ct) value of less than 38 were considered positive.

### 2.3. Whole-Genome Sequencing

RNA extraction was performed using the RNeasy Mini Kit (Qiagen 74104, Hilden, Germany), resulting in a final volume of 40 μL RNA. The hMPV whole genome was enriched from the extracted viral RNA using the Target Capture Kit for hMPV (Baiyi Technology BK-MPXY024, Hangzhou, China). For each sample, reverse transcription was performed first, followed by division into two primer pools for multiplex PCR amplification. Finally, 50 μL of hMPV amplification product was obtained for library construction. After measuring the sample concentration using the Qubit 1X dsDNA HS Assay Kit (Thermo Fisher, MA, USA) on a Qubit 4 fluorometer (Thermo Fisher, MA, USA), 1 μg of DNA from each sample was used for library preparation. According to the manufacturer’s instructions, a maximum of 12 purified samples were barcoded using the Native Barcoding Kit 24 V14 (Oxford Nanopore Technologies SQK-NBD114.24, Oxford, UK), and sequenced for 12 h on a GridION X5 (Oxford Nanopore Technologies, Oxford, UK) using a FLO-MIN114 R10 flow cell (Oxford Nanopore Technologies, Oxford, UK).

### 2.4. Sequence Data Analysis

Sequence assembly of the obtained sequencing data was performed using a bioinformatics analysis platform (Baiyi Technology V5.1, Hangzhou, China). Specifically, assembly parameters included filtering out sequences shorter than 200 bp, trimming 20-nt primer sequences, and excluding regions with a sequencing depth below 10×. Sequence assembly was performed by mapping reads from individual samples to the complete genome sequences of seven reference strains (hMPV A1, A2, A2.1, A2.2.1, A2.2.2, B1, B2). After assembly, individual sequences were exported to NCBI for BLAST analysis (https://blast.ncbi.nlm.nih.gov/, accessed on 2 April 2026). Based on the results, the sequence with the highest similarity was downloaded and used as a reference strain for re-assembly, ultimately yielding the genomic sequence of the sample. The finalized sequences were imported into Nextclade (https://clades.nextstrain.org/, accessed on 2 APR 2026) for subtyping and coverage analysis, followed by phylogenetic tree construction using MEGA (version 12.1.2) to further confirm sample genotypes. The phylogenetic tree was constructed using the maximum likelihood method with 1000 bootstrap replicates and Tamura-Nei model. Phylogenetic analysis incorporated hMPV reference sequences from three sources: definitively typed references spanning seven subtypes obtained from Nextclade (*n* = 5 each for hMPV A1, A2, A2.1, A2.2.1, A2.2.2, B1, and B2), 44 randomly selected sequences retrieved from GenBank (https://www.ncbi.nlm.nih.gov/genbank/, accessed on 2 April 2026), and 139 similar sequences identified through NCBI BLAST searches. All relevant reference sequence files were provided in Supplementary Folder S1. Complete and near-complete hMPV genome sequences were retrieved from GenBank to perform amino acid substitution analysis. After filtering out sequences with less than 97% coverage using Nextclade, sequences belonging to subtypes A2.2.1, A2.2.2, B1, and B2 were extracted and sorted by collection date for amino acid substitution analysis using MEGA (version 12.1.2). Amino acid substitution analysis was performed using HMPV/AUS/136342137/2003/A (KC562224), NL/1/08/A2 (OL794360), NL/2/99/B1 (OL794406), and HMPV/USA/TN-01-28/2001/B (KC562232) as reference strains for A2b1, A2b2, B1, and B2, respectively. High-frequency mutations were defined as those with a base substitution frequency greater than 90% at a specific site. Recombination analysis was conducted using the SimPlot software (Version 3.5.1). Supplementary Folder S2 contains the sequences utilized in this analysis. All obtained 48 sequences have been submitted to GenBank under accession numbers PZ317174 to PZ317221 (Folder S3).

## 3. Results

### 3.1. Epidemiology of hMPV in Jiaxing

**Figure 1:**
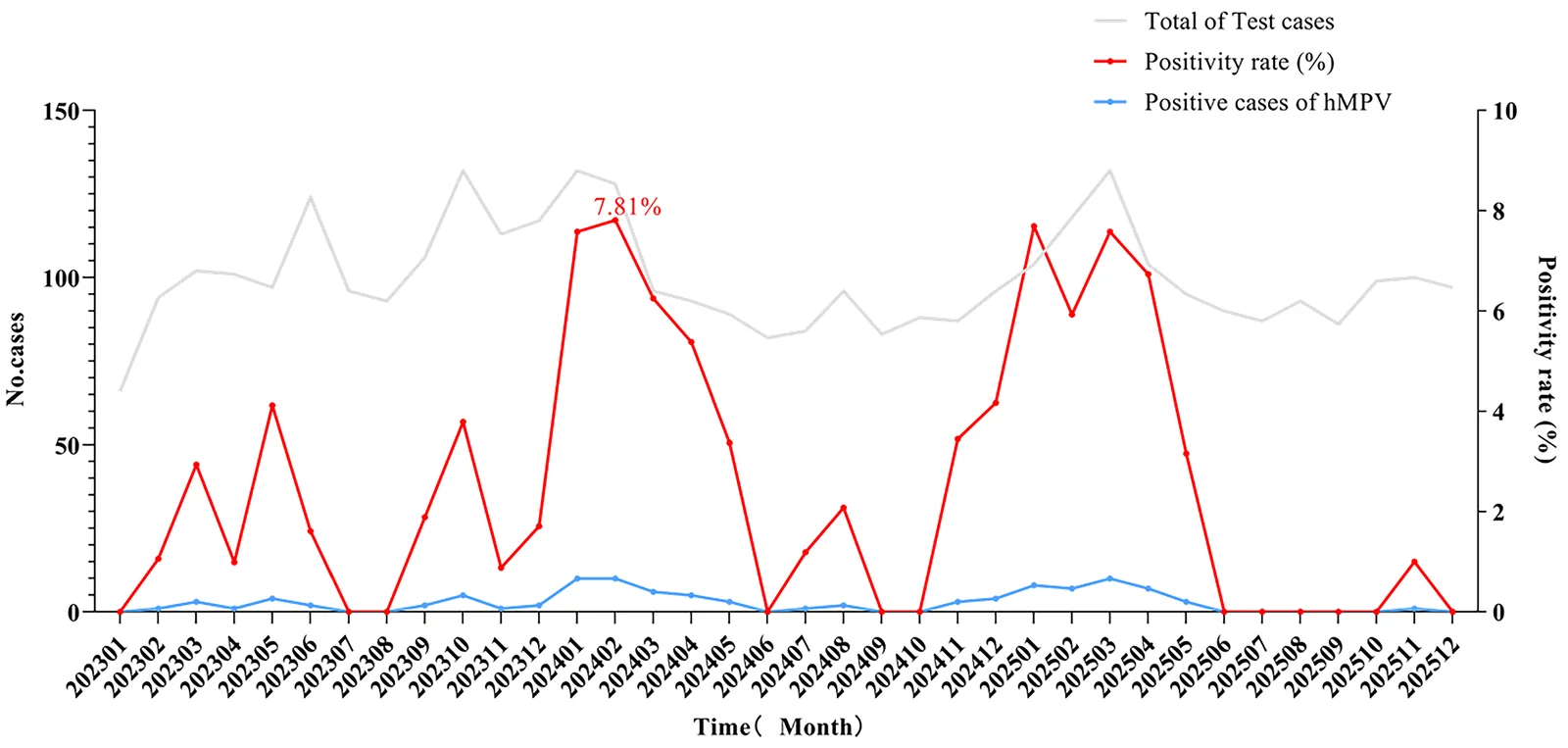
Seasonal and annual distribution of hMPV epidemics in Jiaxing from 2023 to 2025.

From January 2023 to December 2025, a total of 3600 throat swab specimens were collected from ILI patients for respiratory pathogen testing. Through qRT-PCR analysis, 101 specimens (2.81%, 101/3600) tested positive for hMPV. In the 101 specimens, 15 (14.9%, 15/101) were identified with co-infections, including 6 (5.9%, 6/101) with *Haemophilus influenzae*, 2 (2.0%, 2/101) with *Klebsiella pneumoniae*, 3 (3.0%, 3/101) with *Streptococcus pneumoniae*, 1 (1.0%, 1/101) with *Chlamydia pneumoniae*, 2 (2.0%, 2/101) with human rhinovirus, 2 (2.0%, 2/101) with SARS-CoV-2, and 1 (1.0%, 1/101) with human parainfluenza virus type 2. Among the 101 hMPV specimens, 50 (1.39%, 50/3600) were from males and 51 (1.41%, 51/3600) from females, with the chi-square test showing no significant difference in gender distribution (χ^2^ = 0.010, *p* = 0.920). The age distribution of hMPV-positive cases was as follows: 32 cases (0.89%, 32/101) in the 0–5 years group, 20 cases (0.56%, 20/3600) in the 6–14 years group, 31 cases (0.86%, 31/3600) in the 15–59 years group, and 18 cases (0.86%, 31/3600) in the >59 years group. A Pearson chi-square test was used to compare gender composition differences among age groups, and the results showed that the gender distribution across the four age groups was not statistically significant (χ^2^ = 0.387, df = 3, *p* = 0.942, Cramér’s V = 0.062). In Jiaxing, hMPV circulation peaks annually during winter and spring, reaching a maximum monthly positivity rate of 7.81% in February 2024, whereas lower infection rates are observed in summer and autumn (Figure 1).

### 3.2. Genotyping and Phylogenetic Analysis

**Figure 2:**
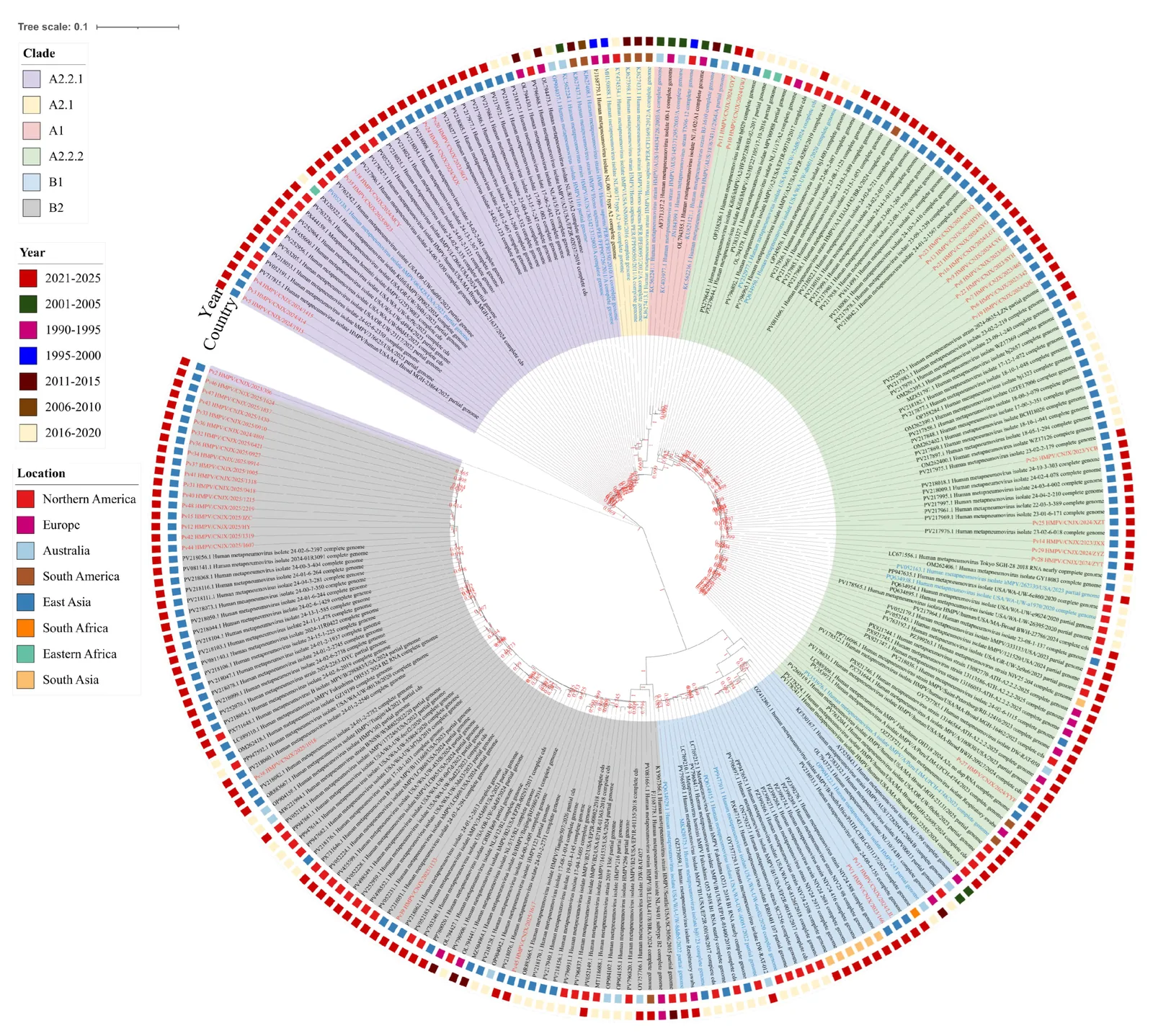
Phylogenetic analysis of hMPV sequences from Jiaxing, 2023–2025. Red font indicates strains submitted from Jiaxing, while blue font denotes sequences with confirmed genotypes in Nextclade. Bootstrap values are displayed in red on the phylogenetic tree.

Among the 101 hMPV-positive samples, 51 were selected for whole-genome sequencing; the remaining samples were excluded from sequencing due to insufficient viral load or degraded RNA quality. A total of 48 hMPV genome sequences were successfully obtained. Based on alignment with the reference sequence hMPV/00-1 (NC_039199) using Nextclade, of the 48 sequences analyzed, one had >97.8% genome coverage, 28 had >98.1%, and 19 had >99.2%. All sequences met the quality threshold and were retained for downstream analysis. All obtained sequences have been submitted to GenBank and assigned the accession numbers PZ317174 through PZ317221. Nextclade typing revealed that of the 48 sequenced isolates, 26 (54.17%) were assigned to hMPV-A and 22 (45.83%) to hMPV-B. More specifically, the distribution was as follows: 8 samples (16.67%) were classified as A2.2.1(A2b1), 18 (37.50%) as A2.2.2(A2b2), 2 (4.17%) as B1, and 20 (41.67%) as B2. Phylogenetic tree analysis confirmed that hMPV subtype assignments were generally concordant with those obtained from Nextclade, except for a single strain, HMPV/CNJX/2023/605, which was identified by sequence alignment as an A2c_111nt-dup_ variant (2.08%, 1/48). In the present study, no A2c_180nt-dup_ variant was detected. In the phylogenetic tree, B1 strains grouped predominantly with those from Beijing, China, whereas A2.2.1, A2.2.2, and B2 strains clustered mainly with strains from the United States and Beijing, China (Figure 2). Despite having circulated in earlier periods, the A1 subtype of hMPV was not detected in the present analysis. No sequence recombination events were detected in the present sequencing analysis.

### 3.3. Subtype Circulation of hMPV

**Figure 3:**
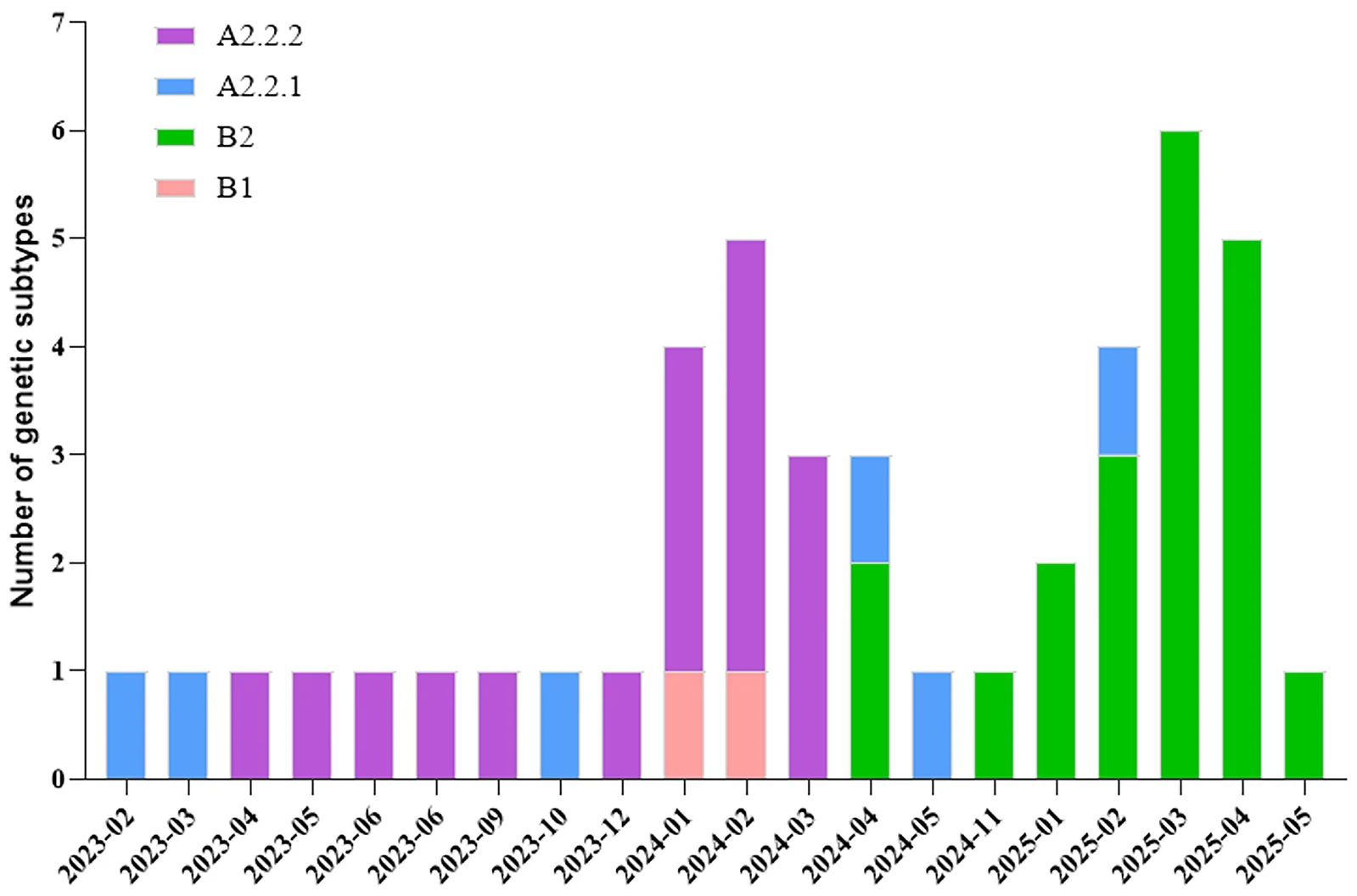
Temporal distribution of hMPV infection subtypes in Jiaxing, 2023–2025.

Different hMPV subtypes circulated in Jiaxing between 2023 and 2025. During 2023, two major hMPV subtypes were in circulation in Jiaxing: subtype A2.2.2 comprised 66.67% (6/9) and subtype A2.2.1 comprised 33.33% (3/9), with subtype A2.2.2 being the dominant strain. In 2024, the spectrum of predominant hMPV subtypes in circulation expanded to four: subtype A2.2.2 comprised 52.63% (10/19), subtype A2.2.1 21.05% (4/19), subtype B2 15.79% (3/19), and subtype B1 10.53% (2/19). Notably, subtype A2.2.2 continued to be the dominant circulating lineage. In 2025, the diversity of circulating hMPV strains reduced to three subtypes: B2 comprised 85% (17/20), A2.2.2 accounted for 10% (2/20), and A2.2.1 represented 5% (1/20), with subtype B2 emerging as the new dominant lineage (Figure 3).

### 3.4. Analysis of Amino Acid Substitutions in hMPV

The hMPV genome contains eight genes, encoding nine structural proteins (N, P, M, F, M1, M2, SH, G, L). A total of 2253 complete or near-complete hMPV sequences, including the 48 obtained from our own sequencing, were downloaded from GenBank for amino acid substitution analysis. After excluding sequences with coverage below 97% via Nextclade analysis, 139 A2.2.1, 857 A2.2.2, 378 B1, and 612 B2 subtype sequences were retained for analysis. In the analysis of amino acid replacements in hMPV proteins, sites with a substitution frequency greater than 90% were defined as high-frequency mutation sites. The collection dates of A2b1 sequences spanned 2003–2025, and we evaluated the frequency of amino acid substitutions after 2016. Among the 57 A2b1 sequences dated 2017 or later, amino acid changes were mainly observed in the G, L, and SH proteins, with the G protein exhibiting the most mutations (*n* = 17), followed by the L protein (*n* = 5). A total of 857 A2b2 sequences were included in the analysis, representing the largest sample size, with collection dates spanning from 2008 to 2025. For this subtype, we focused on assessing amino acid substitution frequencies in sequences obtained after 2019. According to our statistics, a total of 444 A2b2 sequences were collected from 2020 to 2025. Amino acid substitutions occurred predominantly in the G and L proteins, with 6 mutations identified in the G protein and 2 in the L protein. A total of 378 B1 sequences collected between 1991 and 2025 were included in this study, and we mainly assessed the amino acid substitution frequencies in sequences obtained after 2016. Among the 306 post-2016 B1 sequences, high-frequency amino acid mutations were similarly observed in the G, L, and P proteins. In this study, a total of 612 B2 sequences were collected, with submission dates ranging from 2008 to 2025, and we mainly evaluated the frequency of amino acid mutations after 2016. High-frequency amino acid mutations were observed in the F, G, L, P, SH, and M1 proteins of B2 subtype sequences. Notably, among the four subtypes, high-frequency amino acid mutations in the F protein were detected exclusively in the B2 subtype. Detailed information on all amino acid substitutions is provided in Table 1.

## 4. Discussion

HMPV is a respiratory virus newly identified in 2001, but its circulation can be traced back to 1958 [1]. According to a WHO report, the number of hMPV-associated ARTI cases in northern provinces of China has increased since November 2024 (Trends of acute respiratory infection, including human metapneumovirus, in the Northern Hemisphere). Jiaxing is located in southern China; however, the hMPV positivity rate indeed began to rise in November 2024, consistent with the hMPV epidemic trend in China. HMPV infections in Jiaxing display a distinct seasonal pattern, with circulation occurring from December through April and peak activity concentrated between January and March, aligning with the epidemiological trend seen in temperate regions across the Northern Hemisphere [20]. The overall positivity rate of hMPV infection in Jiaxing was 2.81%, falling below the national average of 4.1% reported for China during the 2009–2019 period [21]. The positivity rate of hMPV has been shown to vary by geographic region, with distinct rates observed among individual cities. According to different studies, the overall hMPV positivity rate was 1.6% in Beijing, 4.65% in Shanghai, and 7.14% in Hangzhou [11,22,23]. Despite its geographic proximity to Hangzhou and Shanghai, Jiaxing exhibits a markedly distinct hMPV positivity rate.

HMPV infects individuals across all age groups, but children under 5 years of age and older adults aged 60 years and above are more susceptible, similar to the age distribution observed in respiratory syncytial virus (RSV) infections [24,25]. Among hMPV infection cases in Jiaxing, children under 5 years of age accounted for 31.68% (32/101), and older adults aged 60 and above accounted for 17.82% (18/101), which precisely confirms this trend. However, children accounted for only 0.89% (32/3600) of the total hMPV infections in Jiaxing, markedly below the overall positivity rate. Jiaxing First Hospital is a general hospital, and the sampling included relatively few children because parents of pediatric patients are more inclined to visit the Maternal and Child Health Hospital for care, which likely accounts for the observed discrepancy.

In China, a minimum of five different hMPV lineages (A1, A2b, A2c, B1, and B2) have been identified in circulation; however, the A2b, B1, and B2 lineages constitute the dominant circulating strains [26]. In our study, a total of four hMPV subgenotypes (A2b (A2.2.1), A2c (A2.2.2), B1, and B2) were detected, with no detection of subgenotypes A1 or A2a. Over the same timeframe, the circulating hMPV subtypes in cities near Jiaxing, including Shanghai (predominantly A2b, A2c, and B2) and Hangzhou (predominantly A2b, A2c, B1, and B2), exhibited similar patterns [23,27]. From the perspective of the circulation timing of hMPV epidemics, the prevalence of different subtypes follows certain cyclical patterns. In Jiaxing, A2b/c subtypes dominated circulation from 2023 to 2024; however, in 2025, the B2 subtype emerged as the new predominant lineage. This epidemic trend is similar to that observed in other cities, for example, Hangzhou was dominated by the A2c subtype in 2023, and Beijing was dominated by B2 after July 2024 [22,27]. Phylogenetic analysis revealed that the hMPV strains from Jiaxing belonged to multiple subtypes and grouped predominantly with strains from China and the United States in distinct clades, indicating the possibility of multiple origins for hMPV infections in Jiaxing. While no precise demarcation exists, the overall hMPV epidemic pattern in Jiaxing during 2023–2025 can be generally characterized as an “AAB” model. Since 2015, two mutants (A2c_180nt-dup_ and A2c_111nt-dup_) have been identified in the A2c subtype, characterized by a 180-nucleotide and a 111-nucleotide in-sertion in the G gene, respectively [28,29]. The A2c_111nt-dup_ variant has progressively emerged as the globally predominant strain, a finding further corroborated by hMPV studies conducted in Henan and Beijing. These studies reported that the prevalence of A2c_111nt-dup_ was 40.8% (203/497) in Beijing from 2014 to 2024 and 45.67% (37/81) in Henan from 2017 to 2023 [13,22]. However, in this study, only one A2c_111nt-dup_ strain was detected in Jiaxing, accounting for 5.6% (1/18) of the A2c subgenotype, possibly due to differences in circulating strains.

More than two decades have passed since the initial discovery of hMPV, and the amino acid sequences of contemporary circulating hMPV isolates have diverged considerably from the prototype strain. Subtyping of hMPV is determined by genetic variability in the attachment (G) and fusion (F) glycoproteins, with the G gene representing the most divergent region of the viral genome [18,30]. Compared with previous hMPV strain sequences, the currently circulating strains of the four hMPV subtypes (A2b1/A2b2/B1/B2) harbor a large number of amino acid mutations in the G protein region, while amino acid substitutions in the F protein region occurred only in the B2 subtype (Table 1). The G and F protein regions harbor receptor-binding domains and numerous potential protective antigenic epitopes, and substitutions within these regions may alter viral pathogenicity and facilitate immune evasion. This may represent one of the driving forces behind the transition from hMPV-A to hMPV-B. The T223N and D280N substitutions in the F protein were already mentioned in 2014, but amino acid substitutions in other proteins have not been investigated in relevant studies [31]. The L protein is the longest protein in hMPV strains, and multiple amino acid mutations have also occurred in all four subtypes (A2b1/A2b2/B1/B2). In contrast, other proteins such as M, P, and SH underwent only a small number of amino acid mutations. Based on the analysis of collected sequence data, the timing of amino acid substitutions across hMPV proteins was found to be heterogeneous. According to the sequence submission dates, substitutions in subtypes A2b1, B1, and B2 predominantly occurred after 2016, whereas subtype A2b2 underwent amino acid substitutions after 2019. However, 2016 and 2019 are estimated only based on sequence submission dates and may not reflect the true timing of viral amino acid substitutions, which likely occurred before detection and are challenging to verify. While the nine hMPV proteins serve distinct functions, whether such patterned amino acid replacements influence alterations in hMPV infectivity or virulence is yet to be determined [32].

This study still has certain limitations that warrant consideration when interpreting the findings. First, sampling was restricted to a single tertiary general hospital and limited to febrile influenza-like illness (ILI) cases, which may introduce selection bias. As pediatric patients in Jiaxing frequently seek care at specialized maternal-child hospitals, children aged 0–5 years may be underrepresented in our cohort. Consequently, the observed hMPV positivity rate and age distribution reflect clinically attended infections at this facility rather than the true community-wide burden or population-representative epidemiology. Second, the relatively short sampling period and limited number of sequenced samples preclude robust assessment of long-term seasonal patterns or evolutionary dynamics; notably, we cannot establish definitive temporal nodes for amino acid substitutions, and any reference to specific years should be interpreted solely as descriptive observations within this dataset rather than inferred evolutionary milestones. Third, the lack of additional clinical data limited our ability to analyze the potential association between hMPV subtypes and disease severity. Future multi-center studies incorporating asymptomatic screening, extended surveillance periods, and comprehensive co-infection testing are needed to address these gaps and provide a more complete picture of hMPV transmission and evolution in the region.

## 5. Conclusions

In summary, hMPV is a prevalent respiratory pathogen with circulation dynamics characterized by seasonal fluctuations. Moreover, currently circulating strains have undergone some evolutionary changes compared to historical isolates. In Jiaxing, the dominant hMPV type shifted from A in 2023 to B in 2025, and children under five years and older adults aged ≥60 years represent the major populations of concern. Ongoing monitoring facilitates the real-time assessment of hMPV circulation dynamics and genetic variation, holding significant implications for public health interventions.

## Figures and Tables

**Table 1:** High-frequency amino acid substitution patterns of nine viral proteins (N, P, M, F, M1, M2, SH, G, L) in four hMPV subtypes (A2b1, A2b2, B1, B2).

Subtype	F	G	L	M	M1	M2	N	P	SH
A2b1(57) (Re:KC562224)	/	P69T(100%, 57/57), N74I(96.5%, 55/57), D83Y(96.5%, 55/57), L102F(100%, 57/57), A118T(94.7%, 54/57), I123T(100%, 57/57), S140N(100%, 57/57), N152T(100%, 57/57), S154P(96.5%, 55/57), S155N(96.5%, 55/57), V157T(93.0%, 53/57), R165Q(96.5%, 55/57), V191A(94.7%, 54/57), A/T199V/I (94.7%, 54/57), E204A(94.7%, 54/57), A208T(94.7%, 54/57), M217I(93.0%, 53/57)	T429N(100%, 57/57), R538K(96.5%, 55/57), E540G(96.5%, 55/57), V654I(100%, 57/57), T1388A(91.2%, 52/57)	/	/	S18F (100%, 57/57)	/	V46I(96.5%, 55/57)	S73L(96.5%, 55/57), P132L(96.5%, 55/57), K136E(93.0%, 53/57), E156G(93.0%, 53/57)
A2b2(444) (Re:OL794360)	/	K29R(93.5%, 415/444), L41M/V(93.5%, 415/444), N90S(92.1%, 409/444), K150R(93.2%, 414/444), S162F/L(91.9%, 408/444), R183T(93.5%, 415/444)	T505A(97.7%, 434/444), I772V(92.3%, 410/444)	/	/	/	/	/	/
B1(306) (Re:OL794406)	/	N30Y(98.7%, 302/306), I50V(97.7%, 299/306), P105S(95.8%, 293/306), G116E(99.0%, 303/306), N141H/Y(94.8%, 290/306)	V654I(99.0%, 303/306), V1107A(96.7%, 296/306), V1538A(97.7%, 299/306)	/	/	/	/	E152D(99.3%, 304/306), P277S(98.4%, 301/306)	/
B2(542) (Re:KC562232)	T223N(98.5%, 534/542), D280N(98.7%, 535/542), I392T(98.7%, 535/542), R396Q(98.7%, 535/542), S444N(98.5%, 534/542), K450R(98.2%, 532/542), T521A(91.7%, 497/542)	T55K(90.1%, 492/542), H64L(96.9%, 525/542), D111N(92.4%, 501/542), G116E(97.8%, 530/542), V125I(95.9%, 520/542), T129A(93.2%, 505/542), G183R(90.6%, 491/542)	V55I(98.7%, 535/542), I89T(96.5%, 523/542), S/P123T(94.0%, 509/542), V198I(98.0%, 531/542), T537I(98.5%, 534/542), G760S(97.2%, 527/542), I761T(98.2%, 532/542), I856M(98.0%, 531/542), T960A(97.2%, 527/542), S1118P(96.9%, 525/542)	/	K160R (98.5%, 534/542)	/	/	N44T(98.2%, 532/542), L64P(98.2%, 532/542), K109R(91.61%, 494/542), T120I(97.8%, 530/542)	E14K(98.2%, 532/542), K25N(98.4%, 533/542), H158Y(95.8%, 519/542)

## Data Availability

All obtained sequences have been submitted to GenBank under accession numbers PZ317174 to PZ317221. The accessed URL is https://www.ncbi.nlm.nih.gov/nuccore/ (accessed on 20 April 2026).

## Supplementary Materials

The following supporting information can be downloaded at https://www.mdpi.com/article/10.3390/v18090978/s1: Folder S1: Reference sequences used for phylogenetic tree construction. Folder S2: The amino acid sequences of nine viral proteins—N, P, M, F, M2-1, M2-2, SH, G, and L—from 1986 hMPV strains retrieved from NCBI. Folder S3: The 48 hMPV genome sequences obtained from sequencing.

## Abbreviations

The following abbreviations are used in this manuscript:

hMPV	Human metapneumovirus
China CDC	Chinese Center for Disease Control and Prevention
COVID-19	Corona Virus Disease 2019
ILI	Influenza-like illness
PCR	Polymerase Chain Reaction
NCBI	National Center for Biotechnology Information

## References

[1] van den Hoogen B.G., de Jong J.C., Groen J., Kuiken T., de Groot R., Fouchier R.A., Osterhaus A.D. (2001). A newly discovered human pneumovirus isolated from young children with respiratory tract disease. Nat. Med..

[2] Abrego L.E., Mirazo S., Delfraro A., Franco D., Castillo M., Gaitan M., Castillo J., Moreno B., Pascale J.M., Arbiza J. (2018). Genotypes of human metapneumovirus circulating during 2010–2012 in children from Panama. J. Med. Virol..

[3] Jobe N.B., Rose E., Winn A.K., Goldstein L., Schneider Z.D., Silk B.J. (2025). Human Metapneumovirus Seasonality and Co-Circulation with Respiratory Syncytial Virus—United States, 2014–2024. MMWR Morb. Mortal. Wkly. Rep..

[4] Shakya M., Chu H.Y., Englund J.A., Briggs-Hagen M., Carone M., Kuntz J.L., Lockwood T., Midgley C.M., Schmidt M.A., Starita L. (2025). Epidemiology of Symptomatic Human Metapneumovirus Infection in the CASCADIA Community-Based Cohort—Oregon and Washington, 2022–2024. MMWR Morb. Mortal. Wkly. Rep..

[5] Kulkarni D., Cong B., Ranjini M.J.K., Balchandani G., Chen S., Liang J., Gonzalez Gordon L., Sobanjo-Ter Meulen A., Wang X., Li Y. (2025). The global burden of human metapneumovirus-associated acute respiratory infections in older adults: A systematic review and meta-analysis. Lancet Healthy Longev..

[6] Gray G.C., Capuano A.W., Setterquist S.F., Erdman D.D., Nobbs N.D., Abed Y., Doern G.V., Starks S.E., Boivin G. (2006). Multi-year study of human metapneumovirus infection at a large US Midwestern Medical Referral Center. J. Clin. Virol..

[7] Legrand L., Vabret A., Dina J., Petitjean-Lecherbonnier J., Stephanie G., Cuvillon D., Tripey V., Brouard J., Freymuth F. (2011). Epidemiological and phylogenic study of human metapneumovirus infections during three consecutive outbreaks in Normandy, France. J. Med. Virol..

[8] Edwards K.M., Zhu Y., Griffin M.R., Weinberg G.A., Hall C.B., Szilagyi P.G., Staat M.A., Iwane M., Prill M.M., Williams J.V. (2013). Burden of human metapneumovirus infection in young children. N. Engl. J. Med..

[9] Maggi F., Pifferi M., Vatteroni M., Fornai C., Tempestini E., Anzilotti S., Lanini L., Andreoli E., Ragazzo V., Pistello M. (2003). Human metapneumovirus associated with respiratory tract infections in a 3-year study of nasal swabs from infants in Italy. J. Clin. Microbiol..

[10] CHINA CDC All You Want to Know About Human Metapneumovirus Is Here. https://ivdc.chinacdc.cn/jkzt/kpzs/202306/t20230609_266607.htm.

[11] Du Y., Li W., Guo Y., Li L., Chen Q., He L., Shang S. (2022). Epidemiology and genetic characterization of human metapneumovirus in pediatric patients from Hangzhou China. J. Med. Virol..

[12] Ji L., Chen L., Xu D., Wu X. (2021). Molecular typing and epidemiologic profiles of human metapneumovirus infection among children with severe acute respiratory infection in Huzhou, China. Mol. Biol. Rep..

[13] Xie Z., Zhu Z., Xu J., Mao N., Cui A., Wang W., Wang Y., Zhang Z., Xia B., Wang H. (2024). Seasonal and Genetic Characteristics of Human Metapneumovirus Circulating—Henan Province, China, 2017–2023. China CDC Wkly..

[14] Zhu J., Wu S., Chen Y., Zheng L. (2025). Prevalence and distribution of respiratory pathogens in pediatric acute respiratory infections in Putian, China. BMC Infect. Dis..

[15] Amarasinghe G.K., Arechiga Ceballos N.G., Banyard A.C., Basler C.F., Bavari S., Bennett A.J., Blasdell K.R., Briese T., Bukreyev A., Cai Y. (2018). Taxonomy of the order Mononegavirales: Update 2018. Arch. Virol..

[16] Ye H., Zhang S., Zhang K., Li Y., Chen D., Tan Y., Liang L., Liu M., Liang J., An S. (2023). Epidemiology, genetic characteristics, and association with meteorological factors of human metapneumovirus infection in children in southern China: A 10-year retrospective study. Int. J. Infect. Dis..

[17] Yi L., Zou L., Peng J., Yu J., Song Y., Liang L., Guo Q., Kang M., Ke C., Song T. (2019). Epidemiology, evolution and transmission of human metapneumovirus in Guangzhou China, 2013–2017. Sci. Rep..

[18] Tulloch R.L., Kok J., Carter I., Dwyer D.E., Eden J.S. (2021). An Amplicon-Based Approach for the Whole-Genome Sequencing of Human Metapneumovirus. Viruses.

[19] Fitzner J., Qasmieh S., Mounts A.W., Alexander B., Besselaar T., Briand S., Brown C., Clark S., Dueger E., Gross D. (2018). Revision of clinical case definitions: Influenza-like illness and severe acute respiratory infection. Bull. World Health Organ..

[20] Li Y., Reeves R.M., Wang X., Bassat Q., Brooks W.A., Cohen C., Moore D.P., Nunes M., Rath B., Campbell H. (2019). Global patterns in monthly activity of influenza virus, respiratory syncytial virus, parainfluenza virus, and metapneumovirus: A systematic analysis. Lancet Glob. Health.

[21] Li Z.J., Zhang H.Y., Ren L.L., Lu Q.B., Ren X., Zhang C.H., Wang Y.F., Lin S.H., Zhang X.A., Li J. (2021). Etiological and epidemiological features of acute respiratory infections in China. Nat. Commun..

[22] Li A., Gong C., Wang L., Han Y., Kang L., Hu G., Cao J., Li M., Guan X., Luo M. (2025). Epidemiological and phylogenetic characteristics of human metapneumovirus in Beijing, China, 2014–2024. Signal Transduct. Target. Ther..

[23] Zhu X., Xu M., Lu L., Jia R., Liu P., Bao Z., Xu J. (2026). Prevalence and Molecular Characteristics of Human Metapneumovirus Among Children in Shanghai, China, 2021–2025. J. Med. Virol..

[24] Luo M., Gong C., Zhang Y., Wang X., Liu Y., Luo Q., Li M., Li A., Wang Y., Dong M. (2022). Comparison of infections with respiratory syncytial virus between children and adults: A multicenter surveillance from 2015 to 2019 in Beijing, China. Eur. J. Clin. Microbiol. Infect. Dis..

[25] Divarathna M.V.M., Rafeek R.A.M., Noordeen F. (2020). A review on epidemiology and impact of human metapneumovirus infections in children using TIAB search strategy on PubMed and PubMed Central articles. Rev. Med. Virol..

[26] Feng Y., He T., Zhang B., Yuan H., Zhou Y. (2024). Epidemiology and diagnosis technologies of human metapneumovirus in China: A mini review. Virol. J..

[27] Guo Y.J., Lai Q.R., Song J., Wang Q., Hu B.F., Li Y.L., Wang Y.S., Li W., Ma X.Z. (2025). Clinical and epidemiological characteristics of human metapneumovirus in children in Hangzhou, China. Virol. J..

[28] Saikusa M., Nao N., Kawakami C., Usuku S., Sasao T., Toyozawa T., Takeda M., Okubo I. (2017). A novel 111-nucleotide duplication in the G gene of human metapneumovirus. Microbiol. Immunol..

[29] Saikusa M., Kawakami C., Nao N., Takeda M., Usuku S., Sasao T., Nishimoto K., Toyozawa T. (2017). 180-Nucleotide Duplication in the G Gene of Human metapneumovirus A2b Subgroup Strains Circulating in Yokohama City, Japan, since 2014. Front. Microbiol..

[30] van den Hoogen B.G., Bestebroer T.M., Osterhaus A.D., Fouchier R.A. (2002). Analysis of the genomic sequence of a human metapneumovirus. Virology.

[31] Embarek Mohamed M.S., Reiche J., Jacobsen S., Thabit A.G., Badary M.S., Brune W., Schweiger B., Osmann A.H. (2014). Molecular analysis of human metapneumovirus detected in patients with lower respiratory tract infection in upper egypt. Int. J. Microbiol..

[32] Lianou A., Tsantes A.G., Ioannou P., Bikouli E.D., Batsiou A., Kokkinou A., Tsante K.A., Tsilidis D., Lampridou M., Iacovidou N. (2025). hMPV Outbreaks: Worldwide Implications of a Re-Emerging Respiratory Pathogen. Microorganisms.

